# Recreational Allergic Contact Dermatitis to “Epoxy UV” Resins: A New Source of Exposure to Hidden (Meth)acrylates

**DOI:** 10.1111/cod.70225

**Published:** 2026-07-22

**Authors:** Nadia Raison‐Peyron, Sylvain Caillol, Jean Luc Bourrain, Eva Ibeka, Claire Negrell

**Affiliations:** ^1^ Department of Dermatology Montpellier University Hospital and Montpellier University Montpellier France; ^2^ CNRS Occitanie Est, Pôle Chimie Balard Recherche, ICGM—UMR 5253 Département D2 Montpellier University Montpellier France; ^3^ Department of Pulmonology, Allergology Division Montpellier University Hospital and Montpellier University Montpellier France; ^4^ Desbrest Institute of Epidemiology and Public Health University of Montpellier—INSERM Montpellier France

**Keywords:** allergic contact dermatitis, epoxy UV resin, (meth)acrylates, non‐occupational, nuclear magnetic resonance spectroscopy, recreational

## Abstract

**Background:**

Several cases of contact allergy to epoxy resins used in creative hobbies have been reported in recent years but not to “epoxy UV” resins.

**Objectives:**

To determine which components present in “epoxy UV” resins were responsible for allergic contact dermatitis in three patients who used them to make jewellery as a creative hobby and to describe the clinical and chemical analytical work‐up.

**Methods:**

The three patients with eczematous skin eruptions from “epoxy UV” resin were patch tested with a baseline series and epoxy series and/or additional (meth)acrylates, and in some cases with different benzophenones (patch tests and photo‐patch tests). Proton Nuclear Magnetic Resonance (NMR) of three of the resins used was performed in order to determine their composition.

**Results:**

All patients were shown to be sensitised to (meth)acrylates but not epoxy. Proton NMR could identify 2‐hydroxyethyl methacrylate (2‐HEMA) and an ethoxylated bisphenol A‐based diacrylate oligomer in the three tested resins. For two resins, di(ethylene glycol) diacrylate and poly(propylene glycol) diacrylate were also detected. The resin of one patient could not be analysed because of a technical problem.

**Conclusion:**

Incorrectly labelled “epoxy UV” resins used in recreational activities are a new hidden source of sensitization to (meth)acrylates, not to epoxy compounds tested.

## Graphical abstract

We report three cases of allergic contact dermatitis (ACD) caused by acrylic resins used in creative hobbies, rather than by epoxy resins, despite these products being incorrectly labelled as “epoxy UV” resins.

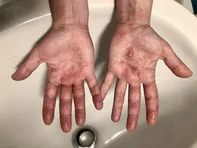

## 1 Introduction

We report three cases of allergic contact dermatitis (ACD) due to acrylic resins used for creative hobbies, and not to epoxy resins as incorrectly labelled (“epoxy UV” resins) on the website where they were purchased. Patch tests were positive for 2‐hydroxyethyl‐methacrylate (HEMA) in the patients whose resins could be analysed, and these resins contained acrylic derivatives, including 2‐HEMA.

## 2 Patients and Methods

### 2.1 Patients

Case 1 A 27‐year‐old female patient, a dental student with no history of atopy and contact allergy, presented with an erythematous vesiculo‐bullous pruritic eruption of both hands, extending secondarily to the forearms, thighs, and right ear probably of hand‐borne origin (Figure 1).

**FIGURE 1:**
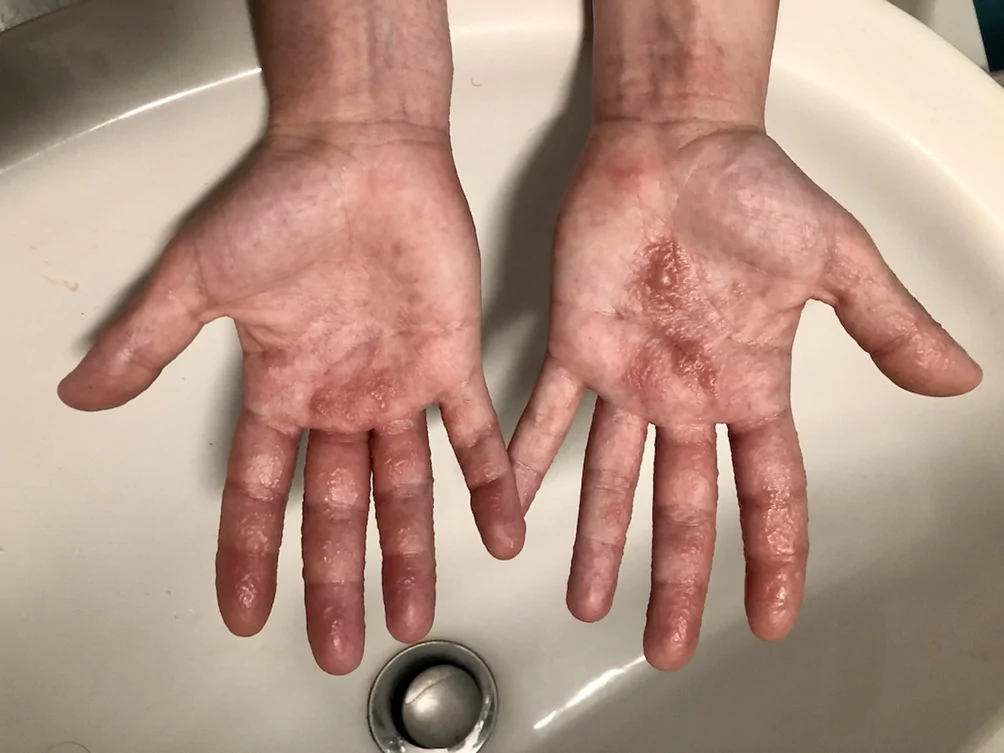
Acute erythematous and vesiculo‐bullous lesions of the hands.

Lesions appeared 2 days after she made, without protective gloves, for the first time, costume jewellery on which a liquid UV resin (MZJAUVF with no other information) purchased on the leading e‐commerce website in France had been applied and then polymerised under a 6‐watt UV lamp supplied with the bottle. This resin was listed as a “epoxy UV” resin on the website. However, no labelling regarding the exact composition of the resin was found on the bottle or packaging.

The eruption gradually resolved in 3 weeks under corticosteroid cream.

Patch testing was performed with IQ Ultra Chambers occluded for 2 days with Opertape (Iberhospitex, Innovative Health Technologies, Barcelona, Spain). Patch tests with the European baseline series along with additional series from the REVIDAL‐GERDA group (Réseau de Vigilance en Dermato‐Allergologie du Groupe d'Etude et de Recherche en Dermato‐Allergologie), the epoxy series (Chemotechnique Diagnostics, Vellinge, Sweden), except for the 1,4‐butanediol diglycidyl ether (1,4‐BDDE) (CAS No 952425‐79‐8) from Allergeaze, SmartPractice Canada, and with the “epoxy UV” resin tested at 0.1% and 1% in petrolatum (pet) were carried out and read on day (D)2 and D3 according to the ESCD guidelines [1]. We completed the assessment with photo patch testing with 3 and 10‐benzophenone (BP) 10%pet, 4‐BP 2%pet and with the resin at both dilutions, irradiated with 5 J/cm^2^ of fluorescent UVA (UVA L 801; Herbert Waldmann GmbH& Co KG).

Further photopatch testing was carried out, using the same protocol, with well‐known photoinitiators in the industry (UV‐curable resins) such as the unmodified BP (CAS No. 119‐61‐9), the 4 methyl‐BP (CAS No. 134‐84‐9) and the 1‐hydroxy‐cyclohexyl‐phenyl‐ketone (1‐HCPK) (CAS No. 947‐19‐3) from Sigma‐Aldrich, Darmstadt, Germany (all diluted in 5%, 0.5% and 0.05%pet).

Case 2 A 25‐year‐old female patient with past history of asthma and allergic rhino conjunctivitis presented with recurrent episodes of erythematous, vesicular, and pruritic lesions on the palms and lateral aspect of the fingers, a few months after starting to create handmade jewellery at home as a business.

She used a UV cured liquid resin called “epoxy UV” purchased under the name CIANBIFN on the same e‐commerce website as in Case 1 and worn nitrile gloves during each creation process. Under corticosteroid cream, the lesions partially improved. However, she had to stop this activity due to many relapses of dermatitis when she was in contact with the resin.

Patch testing was conducted with the European baseline series, the epoxy series, and the nail acrylates series. The resin itself was not tested.

Case 3 A 61‐year‐old female patient with a history of type 2 diabetes mellitus, perennial rhinitis, overactive bladder treated with transcutaneous electrical nerve stimulation (TENS), two total hips and one total knee prosthesis was referred for the evaluation of pruritic, scaly, and fissured lesions on the finger pulps of both hands. She handled resins to make jewellery as creative hobbies for 1 year, using two “epoxy UV” resins and a UV lamp, both purchased on another popular e‐commerce website, without any information on the product's composition on the site and on the packaging. She mentioned that she had localized pruritus around the electrodes of TENS before she began to make resin jewellery. In a second time, the lesions spread to the back of the hands, the thighs, probably of hand‐borne origin and to the lateral aspect of the neck when she wore the resin earrings she had made.

Patch tests were performed with the European baseline series, the epoxy and nail acrylates series. Photo‐patch tests were performed with BPs and 1‐HCPK as in Case 1. The resin itself was not tested.

### 2.2 Chemical Analyses

#### 2.2.1 Nuclear Magnetic Resonance (NMR) Spectroscopy

NMR samples were prepared with deuterated chloroform (CDCl3) as solvent and the analyses were performed using a Bruker Avance 400 MHz spectrometer. The structure of the resins was determined by proton NMR. External references were timethylsilane (TMS) and shifts were given in ppm.

## 3 Results

### 3.1 Patch Test Results

All relevant patch test results are summarized in Table 1. Two patients (Case 1 and 3) reacted strongly to 2‐HEMA (+++) (CAS No. 868‐77‐9) corresponding to compound M1. The patients in Cases 2 and 3 also reacted to other (meth)acrylates. It should be noted that Case 1 was not tested with the (meth)acrylates series because they were not initially suspected. The resin, which was tested only in Case 1, showed a strong positive reaction (++) with marked photoaggravation (+++) (Figure 2). Interestingly, this patient also reacted to 1,4‐butanediol diglycidyl ether (++). Neither of the two tested patients showed sensitization or photosensitization to benzophenones (BP).

**TABLE 1:** Main results of patch and photo‐patch tests.

Lecture on D3	European standard series	Epoxy series	Acrylates series	Substituted BP of the PPT series (3‐, 4‐ and 10)	Unmodified BP NI I	MBP NI I	HCPK NI I	Resin itself NI I
Patient 1	2‐HEMA +++	1,4‐BDDGE ++	NT	Neg	Neg	Neg	Neg	++ +++
Patient 2	Neg	Neg	EGDMA ++	NT	NT	NT	NT	NT
			2‐HEA ++					
			EA ++					
Patient 3	2‐HEMA +++	Neg	EGDMA ++	Neg	Neg	Neg	Neg	NT
			2‐HEA++					
			EA++					
			2‐HPMA+, DEGDA+					

Abbreviations: 1,4 BDDGE, 1,4‐butanediol diglycidyl ether 0.25%pet; 2‐HEA, 2‐hydroxyethyl acrylate 0.1%pet; 2‐HEMA, 2‐hydroxyethyl methacrylate 2%pet; 2‐HPMA, 2‐hydroxypropyl methacrylate 2%pet; 4‐MBP, 4‐methyl‐benzophenone; BP, benzophenone; DEGDA, diethylene glycol diacrylate 0.1%pet; EA, ethyl acrylate 0.1%pet; EGDMA, ethylene glycol dimethacrylate 2%pet; HCPK, 1‐hydroxy‐cyclohexyl‐phenyl ketone, all three at 0.05, 0.5 and 5%pet; I, irradiated; NI, non irradiated; PPT, photo‐patch tests.

**FIGURE 2:**
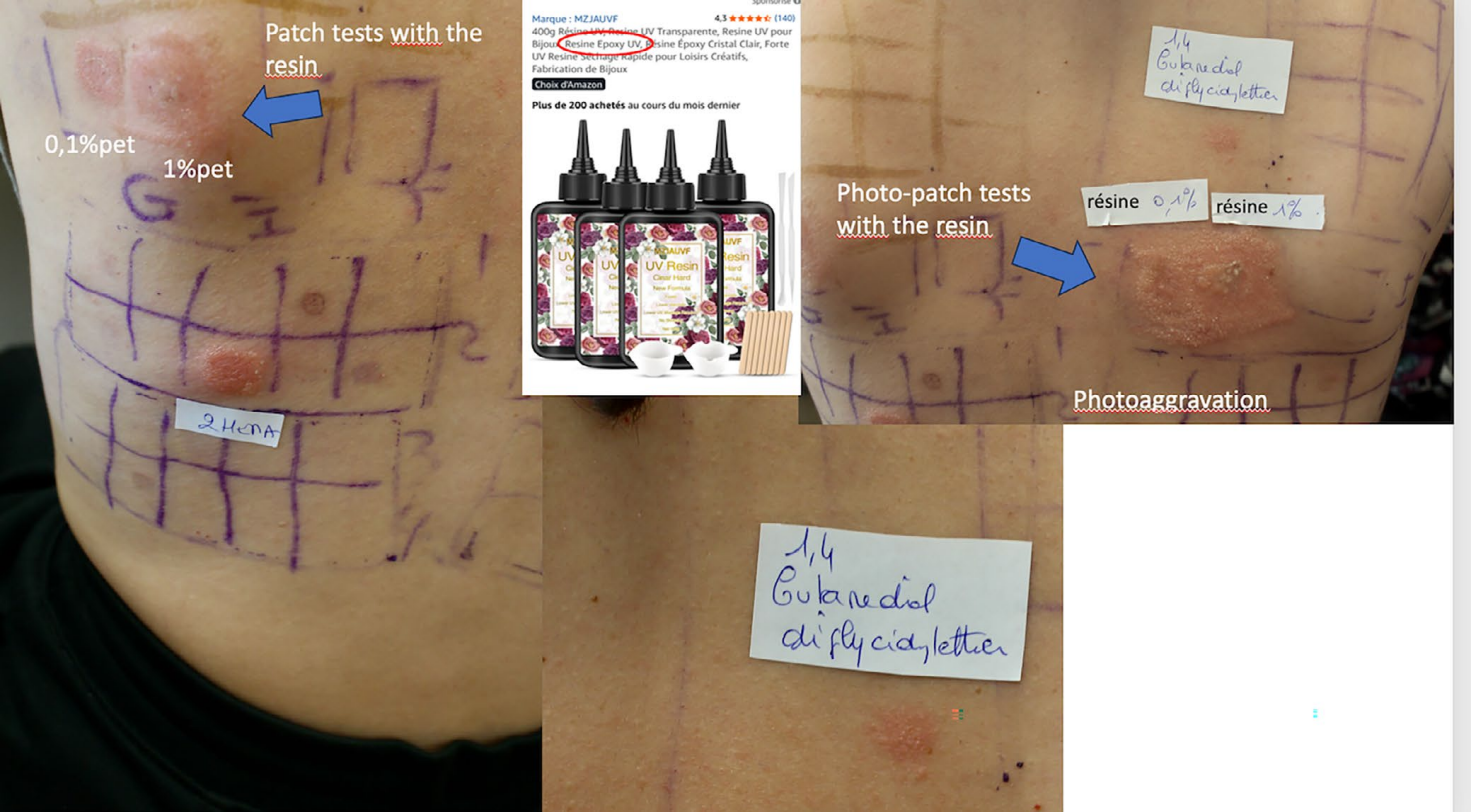
Patch tests results for patient in Case 1 on D3: 2‐HEMA 2%pet +++, resin ++ 0.1 pet and 1%pet, 1,4 BDDGE 0.25%pet ++. Photopatch tests results with the resin ++ 0.1%pet, +++1%pet.

### 3.2 Chemical Analyses

M1, M2, M3 and M4 are internal labels used to designate the compounds identified in the proton NMR analysis and shown in Figure 3.

**FIGURE 3:**
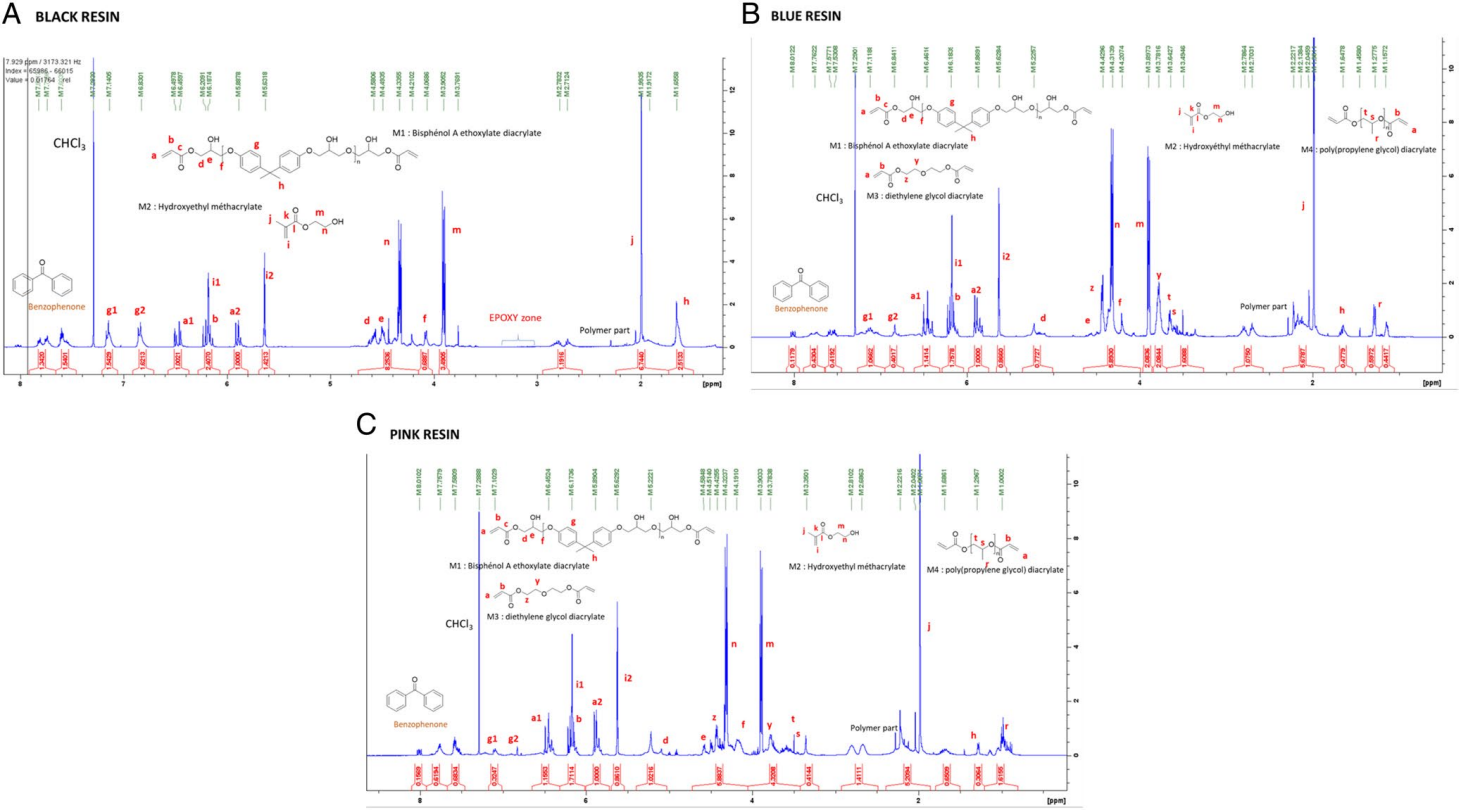
Results of ^1^H NMR spectroscopy of the three resins. Presence of HEMA and BPA in all the resins and of di(ethylene glycol) diacrylate and poly(propylene glycol) diacrylate in variable proportions in the “blue” and “pink” resin.

#### 3.2.1 Case 1

Based on the proton NMR spectrum of the resin (named black resin) and its infrared analysis, the presence of two main monomers that undergo UV‐induced polymerization was identified: a bisphenol A ethoxylate diacrylate (CAS No. 64401–02‐1) which is a difunctional diacrylate monomer, derived from diglycidyl ether of bisphenol A which provides rigidity and crosslinking to the resin (M1) and the second monomer, HEMA (M2), which acts as a reactive diluent: it reduces the viscosity of the resin and polymerises under UV irradiation simultaneously with the first difunctional monomer. The mixture consists of these two monomers in approximately a 40/60 ratio (Figure 3A).

In the region corresponding to the characteristic signals of the epoxy functional group, no peaks were observed, suggesting its absence. The presence of a benzophenone derivative was also detected; this compound acts as a photoinitiator, triggering the polymerization reaction under UV exposure, and was present in relatively high concentration. Broad peaks at 2.78–2.71 ppm, as well as around 1.9 ppm, were associated with partial polymerization of acrylate functionalities occurring during storage at room temperature. Some signals remained unassigned and likely corresponded to additives; further analyses would be required for their identification.

#### 3.2.2 Case 2

The resin was unavailable for analyses.

#### 3.2.3 Case 3

Two different resins were analysed. Regarding the resin with the blue label, the proportion of ethoxylated bisphenol A diacrylate was lower, and two difunctional monomers were present in the formulation: di(ethylene glycol) diacrylate (M3) (CAS No. 4074‐88‐8) and poly(propylene glycol) diacrylate (M4) (CAS No. 52496‐08‐9). These two difunctional monomers exhibited similar reactivity to the aromatic monomer but had different viscosities, allowing adjustment of this parameter in the final product. The monomer ratios were approximately 60/20/12/8 for M2/M3/M1/M4, respectively (Figure 3B).

For the resin with the pink label, a decrease in the content of aromatic diacrylate was observed, while the proportion of the reactive diluent based on HEMA remained high, around 60%. Among the difunctional monomers present, the aromatic diacrylate content was reduced, with a distribution of M3/M1/M4 of approximately 30/5/5. In this resin, in addition to peaks indicating the onset of polymerization of certain acrylate monomers, a broad peak around 1 ppm was observed, characteristic of a CH_3_ group (Figure 3C).

## 4 Discussion

ACD to epoxy resin systems, well‐known occupational allergens, is increasingly reported in non‐occupational settings, particularly in do‐it‐yourself activities. In these cases, online‐purchased epoxy resins are used for various creative purposes, affecting both adults and children [2, 3, 4, 5].

Epoxy resins consist of a resin and hardener that polymerise at room temperature. The main sensitizers are diglycidyl ether derivatives of bisphenol A (DGEBA) or bisphenol F (DGEBF) [6, 7, 8].

In industrial settings, some epoxy systems can also be UV‐cured via cationic polymerization in the presence of photoinitiators, leading to rapid crosslinking. These UV‐curable systems are used in adhesives, coatings, inks, 3D printing, and dental materials [9, 10, 11, 12]. Cases of ACD related to epoxy acrylate‐containing systems have been reported, mainly in occupational exposure [13, 14].

The resins that we analysed and which are commercially marketed as “epoxy UV” resins are different because of the absence of the epoxy functional group. We initially thought they were photopolymerizable epoxy resins, but they turned out not to be that type of resin. In reality, they are acrylate‐based and cure via UV‐induced radical polymerization such as for artificial nails. Proton NMR analysis identified bisphenol A ethoxylate diacrylate, an ethoxylated bisphenol A‐based diacrylate oligomer, and (meth)acrylates but no functional epoxy group. Bisphenol A (BPA) is synthesized from phenol and acetone, while DGEBA is produced by the reaction of BPA with epichlorohydrin. In contrast, BPA‐based acrylate resins are not necessarily derived from DGEBA. They can be obtained either by direct acrylation of BPA derivatives, yielding BPA diacrylate, or after ethoxylation of BPA, leading to bisphenol A ethoxylate diacrylate. Consequently, these materials do not contain epoxy functionalities originating from DGEBA, and no residual epoxy groups were detected in the analysed resin samples. Therefore, we believe that the term “epoxy UV” resin is not appropriate for describing these systems.

Moreover, to our knowledge, bisphenol A ethoxylate diacrylate has not been reported as a skin sensitizer.

Acrylates and methacrylates ((meth)acrylates) are esters of acrylic and methacrylic acid that polymerise into solid resins through various curing processes, including chemical initiation, UV or heat exposure [15, 16]. They are well‐known causes of contact allergy and ACD, with low‐molecular weight monomers acting as the main sensitizers [17]. Fully cured resins are generally non‐reactive, although incomplete polymerization may leave residual allergenic monomers.

Occupational exposure to (meth)acrylates is well documented, particularly in dentistry, printing, painting, and adhesive‐related industries [18, 19, 20, 21]. More recently, a marked increase in ACD has been reported in relation to nail cosmetic products, leading to the inclusion of 2‐HEMA in the European baseline patch test series in 2019 [22, 23, 24].

Although 2‐HEMA was detected in all tested resins in our study, to our knowledge no cases of ACD related to (meth)acrylates in arts and crafts products have been reported [25, 26]. In the EU, HEMA is restricted to professional‐use nail products with mandatory allergen labelling, and non‐compliant cosmetic products have been prohibited since September 2021 [27]; however UE cosmetics legislation does not apply to hobby items and related products.

It is important to emphasise that the second patient who was sensitised to several (meth)acrylates did not have a positive test for 2‐HEMA, which seems to suggest that it may not be a reliable marker for detecting allergies to this type of product. Unfortunately, the resin used by this patient could not be analysed. In the two resin samples from the third patient, di(ethylene glycol) diacrylate (M3) and poly(propylene glycol) diacrylate were also identified, which are two difunctional monomers. This patient had positive patch tests to di(ethylene glycol) diacrylate. We cannot rule out the possibility that this patient was sensitised through the adhesive of the TENS electrodes, which may contain (meth)acrylates [28].

It would have been desirable to test these other identified components, namely M1, M3, and M4, in order to identify the true contact allergens in these patients. Unfortunately, we were unable to obtain these substances for testing. Benzophenone derivatives used as photoinitiators were also detected in the three analysed resin samples; however, neither of the two tested patients showed sensitisation or photosensitisation to BP.

The three patients were highly sensitised to (meth)acrylates and only the first patient who is a dental student was sensitised to 1,4 BDDGE, a crosslinking agent and viscosity modifier for epoxy systems which is used sometimes in dentistry [29, 30]. It cannot be excluded that the patient was sensitised to 1,4 BDDGE because of occupational exposure.

This patient presented with acute vesicular and bullous eczema, whereas the other patients had significantly less severe lesions. Furthermore, we performed testing with the resin itself only in this patient and observed strong positive tests with photoaggravation. We do not have a precise explanation for this result. It may be that the amount of product placed in the irradiated test chamber was higher. Benzophenones do not appear to be implicated, given the negative photopatch tests. Likewise, it is unlikely that new acrylates are formed during the photopolymerization step. On the contrary, polyacrylates are formed (Figure S1).

It's important to point out that none of them used artificial nail products.

Regarding these different types of resins used for arts and crafts, many advantages of “epoxy UV” resins compared to traditional two‐part (hardener‐based) epoxy resins are alleged on the commercial websites: instant curing under UV light instead of several hours or even days for traditional epoxy resins, no mixing is required, which reduces the risk of incorrect ratios and bubbles and they are suitable for thin coating and small artworks. All these resins were ordered online, and their formulation was not mentioned. Even worse, they were presented on world‐famous online shopping sites as “ideal products for creating art at home or as a gift for family, children and friends”. Most consumers using these resins don't wear protective gloves. It is therefore a new source of exposure to strong allergens, i.e., acrylates especially among young people, alongside the growing use of semi‐permanent nail polishes cured under UV [31].

It is also important to underline that the name of the resins such as “MZJAUVF” is not an officially known or registered company, but a name used for products sold on e‐commerce sites, often without a real brand or clearly identified manufacturer. Therefore, it's hard to identify who is responsible, so authorities can't easily act, and consumers may have no direct recourse. This highlights the need for carrying out awareness campaigns to educate patients. Some regulation of such products sold on e‐commerce sites which contain strong allergens (epoxy and/or (meth)acrylates) marketed to the public including young people for recreational activities would be necessary.

## Author Contributions

**Nadia Raison‐Peyron:** conceptualization, investigation, methodology, validation, visualization, writing – original draft, writing – review and editing, supervision. **Jean Luc Bourrain:** investigation, visualization, writing – review and editing, resources. **Sylvain Caillol:** investigation, methodology, formal analysis, visualization. **Eva Ibeka:** investigation, writing – review and editing, visualization. **Claire Negrell:** investigation, funding acquisition, supervision, writing – review and editing, formal analysis, validation, methodology.

## Conflicts of Interest

The authors declare no conflicts of interest.

## Supporting information


**Figure S1:** Schematic view of the acrylate polymer network formed after photopolymerization.

## Data Availability

The data that support the findings of this study are available from the corresponding author upon reasonable request.
