# Supplementary material for: Recreational Allergic Contact Dermatitis to “Epoxy UV” Resins: A New Source of Exposure to Hidden (Meth)acrylates
Source: Contact Dermatitis. 2026 Jul 22;95(5):475–81. doi: 10.1111/cod.70225 (PMC13648317; doi:10.1111/cod.70225)
Supplement: Supplementary file 1 [file COD-95-475-s001.docx]

**Supplementary Figure 1** : Schematic view of the acrylate polymer network formed after photopolymerization
